# ﻿Two new species of *Neohelicosporium* (Tubeufiaceae, Tubeufiales) from freshwater and terrestrial habitats in China

**DOI:** 10.3897/mycokeys.118.151514

**Published:** 2025-05-19

**Authors:** Xiao-Yan Ma, Yong-Zhong Lu, Lei He, Dan-Dan Song, Jian Ma

**Affiliations:** 1 School of Food and Pharmaceutical Engineering, Guizhou Institute of Technology, Guiyang 550003, China Guizhou Institute of Technology Guiyang China; 2 Guizhou Academy of Agricultural Sciences, Guiyang 550025, China Guizhou Academy of Agricultural Sciences Guiyang China; 3 College of Brewing Engineering, Moutai Institute, Renhuai 564507, China College of Brewing Engineering Renhuai China; 4 Guizhou Industry Polytechnic College, Guiyang 550008, China Guizhou Industry Polytechnic College Guiyang China

**Keywords:** 2 new species, asexual morph, Dothideomycetes, phylogeny, saprobic fungi, taxonomy

## Abstract

*Neohelicosporium* species are a group of helicosporous hyphomycetes with the potential to produce novel bioactive natural compounds. During our investigation on helicosporous hyphomycetes, six isolates were collected from freshwater and terrestrial habitats in the tropical and subtropical regions of southern China. Based on multi-gene phylogenetic analyses (LSU-ITS-*tef*1-α-*rpb*2) and morphological comparisons of *Neohelicosporium* taxa, two new species (*N.guizhouense* and *N.wuzhishanense*) are introduced. Detailed micro-morphological descriptions, illustrations, and DNA molecular data are provided for the newly introduced species to confirm their taxonomic placements.

## ﻿Introduction

*Neohelicosporium* was established by Lu et al. (2018a) based on morphological characteristics and phylogenetic evidence. In their study, five new species, *Neohelicosporiumaquaticum*, *N.guangxiense*, *N.hyalosporum*, *N.parvisporum* (the type species), and *N.thailandicum*, were collected from submerged decaying wood in southern China and Thailand (Lu et al. 2018a). Jayasiri et al. (2017) reported the first asexual-sexual morph of *Neohelicosporiumfusisporum*, collected from decaying fruit of a Malvaceae species in Thailand. Subsequently, Lu et al. (2018b) reassessed the order Tubeufiales based on DNA sequence data and morphological characteristics, introducing seven new species and nine new combinations within *Neohelicosporium* (Morgan 1892; Penzig 1897; Linder 1929; Moore 1954; Rao and Rao 1964; Matsushima 1971, 1980; Rossman 1979, 1987; Chouhan and Panwar 1980; Goos 1985, 1986, 1989; Ho et al. 2001; Tsui et al. 2001, 2006; Cai et al. 2006; Zhao et al. 2007; da Cruz et al. 2009; Promputtha and Miller 2010; Singh and Singh 2016; Jayasiri et al. 2017; Kuo and Goh 2018; Lu et al. 2018a). Dong et al. (2020) introduced another species, *Neohelicosporiumsubmersum*, collected from a freshwater habitat in Thailand. Subsequent studies reporting novel *Neohelicosporium* species are listed chronologically: *N.suae* collected from submerged decaying wood in China (Li et al. 2022); *N.bambusicola* collected from dead culms of bamboo in Thailand (Tian et al. 2022); *N.hainanensis* collected from decaying wood in a terrestrial habitat in China (Lu et al. 2022); *N.terrestris* collected from a dead leaf petiole of *Musa* sp. in Thailand (Samarakoon et al. 2024); *N.guineensis* and *N.xishuangbannaensis* collected from rotting petioles of *Elaeisguineensis* in China (Xiong et al. 2024); and *N.baihualingense*, *N.hainanense*, *N.jianfenglingense*, and *N.latisporum* collected from freshwater and terrestrial habitats in China (Ma et al. 2024b).

To date, based on morphological and/or molecular data, *Neohelicosporium* contains 33 species, with four species (*N.myrtacearum*, *N.nizamabadense*, *N.sympodiophorum*, and *N.vesiculiferum*) lacking molecular data. *Neohelicosporium* species are widely distributed across freshwater and terrestrial habitats in Australia, Belgium, Brazil, Canada, China, Cuba, Germany, Honduras, India, Indonesia, Japan, New Zealand, Panama, Peru, the Solomon Islands, South Africa, Thailand, Venezuela, and the USA (Morgan 1892; Penzig 1897; Linder 1929; Moore 1954; Rao and Rao 1964; Matsushima 1971, 1980; Rossman 1979, 1987; Chouhan and Panwar 1980; Goos 1985, 1986, 1989; Ho et al. 2001; Tsui et al. 2001, 2006; Cai et al. 2006; Zhao et al. 2007; da Cruz et al. 2009; Promputtha and Miller 2010; Singh and Singh 2016; Jayasiri et al. 2017; Kuo and Goh 2018; Lu et al. 2018a, 2018b; Dong et al. 2020; Li et al. 2022; Tian et al. 2022; Yang et al. 2023; Zhang et al. 2023a, 2023b; Samarakoon et al. 2024; Ma et al. 2023, 2024b). Among them, 28 *Neohelicosporium* species exhibit a helicosporous asexual morph, 4 species display both asexual and sexual morphs, and one species (*N.terrestris*) produces only the sexual morph (Promputtha and Miller 2010; Jayasiri et al. 2017; Lu et al. 2018b; Samarakoon et al. 2024). The asexual morph of *Neohelicosporium* is characterized by white, light pink, or pale brown colonies; short and solitary conidiophores that are mostly branched and gregarious; mono- to polyblastic conidiogenous cells; and guttulate, hyaline, helicoid conidia (Lu et al. 2018a, 2018b; Dong et al. 2020; Li et al. 2022; Tian et al. 2022; Yang et al. 2023; Ma et al. 2024b). Its sexual morph is depicted by globose to subglobose, spherical or ellipsoidal-ovate, pale brown to yellow or dark brown to black ascomata, 8-spored or 2–4–6-spored, bitunicate asci, and fusiform, hyaline ascospores (Promputtha and Miller 2010; Jayasiri et al. 2017; Lu et al. 2018b; Samarakoon et al. 2024).

In this study, six helicosporous isolates representing two distinct species were collected from freshwater and terrestrial habitats in southern China. Based on comprehensive morphological descriptions and illustrations, as well as multi-gene phylogenetic analyses, two novel species, namely *Neohelicosporiumguizhouense* and *N.wuzhishanense*, are introduced.

## ﻿Materials and methods

### ﻿Sample collection and specimen examination

Specimens were collected from freshwater and terrestrial habitats between December 2021 and April 2022 in Qiannan Buyi and Miao Autonomous Prefecture, Sandu Shui Autonomous County, Guizhou Province, and Ledong Li Autonomous County and Wuzhishan City, Hainan Province, China, and the important collection details were noted (Rathnayaka et al. 2024). Once the specimens were transported to the laboratory, the specimens from freshwater habitats were incubated at room temperature and maintained in a moist environment for 1–2 weeks. Fungal colonies on the surface of the natural substrates, including conidiophores, conidiogenous cells, and conidia, were examined using a stereomicroscope (SMZ-168, Nikon, Japan). Photographs were taken with an ECLIPSE Ni compound microscope (Nikon, Tokyo, Japan) equipped with a Canon 90D digital camera.

### ﻿Isolation and material deposition

Single spore isolations were performed following the method described by Senanayake et al. (2020). The germinated coiled conidia were aseptically transferred onto fresh potato dextrose agar (PDA) plates as described by Senanayake et al. (2020). The fungal mycelia were cultured on PDA medium at a constant temperature of 25 °C for 33–41 days. During incubation, colony characteristics such as shape, color, size, margin, and elevation were systematically documented.

Dried fungal specimens were deposited in the Herbarium of Kunming Institute of Botany, Chinese Academy of Sciences (Herb. HKAS), Kunming, China, and the Herbarium of Guizhou Academy of Agriculture Sciences (Herb. GZAAS), Guiyang, China. Pure cultures were deposited at the Guizhou Culture Collection (GZCC), Guiyang, China. Descriptions of the new taxa were uploaded to the Faces of Fungi webpage, as per the guidelines provided by Jayasiri et al. (2015). The new species were registered in the MycoBank database (https://www.mycobank.org/), and MycoBank numbers were obtained.

### ﻿DNA extraction, PCR amplification, and sequencing

Fresh mycelia were scraped using sterilized toothpicks. Genomic DNA was extracted using the Biospin Fungus Genomic DNA Extraction Kit (BioFlux, China), following the manufacturer’s protocol. Primer pairs LR0R/LR5 (Vilgalys and Hester 1990), ITS5/ITS4 (White et al. 1990), EF1-983F/EF1-2218R (Rehner and Buckley 2005), and fRPB2-5F/fRPB2-7cR (Liu et al. 1999) were used to amplify LSU, ITS, *tef*1-α, and *rpb2* sequence fragments, respectively. The PCR amplification reactions were performed according to the protocol by Ma et al. (2024a). The PCR products were purified and sequenced by Qingke Biotechnology, Chongqing, China.

### ﻿Phylogenetic analyses

Newly generated sequences were verified and assembled using BioEdit v. 7.0.5.3 (Hall 1999) and SeqMan v. 7.0.0 (Swindell and Plasterer 1997). Additional sequences used in this study were obtained from GenBank (Table 1; https://www.ncbi.nlm.nih.gov/). Alignments for the LSU, ITS, *tef*1-α, and *rpb*2 datasets were performed using MAFFT v. 7.473 (https://mafft.cbrc.jp/alignment/server/, Katoh et al. 2019), and were visually checked and converted to the nexus format using AliView v. 1.27 (Daniel et al. 2010; Larsson 2014). Subsequently, trimAl v. 1.2 software was used to trim each gene dataset (Capella-Gutiérrez et al. 2009). The LSU, ITS, *tef*1-α, and *rpb*2 datasets were then concatenated using SequenceMatrix v. 1.7.8 (Vaidya et al. 2011).

**Table 1.** List of taxa analyzed in this study, along with their corresponding GenBank accession numbers.

Taxon	Strain	GenBank Accession numbers			
		LSU	ITS	*tef*1–α	*rpb2*
*Acanthohelicosporaaurea*	GZCC 16-0060	KY321326	KY321323	KY792600	MF589911
*A.guianensis*	UAMH 1699	AY856891	AY916479	–	–
*Neohelicosporiumabuense*	CBS 101688	–	AY916470	–	–
*N.acrogenisporum*	MFLUCC 17-2019^T^	MH558871	MH558746	MH550937	MH551069
*N.aquaticum*	GZCC 23-0342	PP639461	PP626605	PP596362	PP596488
*N.aquaticum*	MFLUCC 17-1519^T^	MF467929	MF467916	MF535242	MF535272
*N.astrictum*	MFLUCC 17-2004^T^	MH558872	MH558747	MH550938	MH551070
*N.aurantiellum*	GZCC 23-0135	PP639462	PP626606	–	–
*N.aurantiellum*	GZCC 23-0414	PP639463	PP626607	–	–
*N.aurantiellum*	ANM 718	GQ850485	GQ856140	–	–
*N.baihualingense*	CGMCC 3.25547^T^	–	PP626608	PP596363	PP596489
*N.baihualingense*	GZCC 23-0235	PP639465	PP626609	PP596364	PP596490
*N.bambusicola*	MFLUCC 21_0156^T^	OL606146	OL606157	OL964517	OL964523
*N.ellipsoideum*	GZCC 22-2072	PP639466	PP626610	PP596365	–
*N.ellipsoideum*	MFLUCC 16-0229^T^	MH558873	MH558748	MH550939	MH551071
*N.fluviatile*	MFLUCC 15-0606^T^	OP377957	–	OP473050	OP473111
*N.fluviatile*	HKUCC 10235	AY849942	–	–	–
*N.fusisporum*	MFUCC 16-0642^T^	MG017613	MG017612	MG017614	–
*N.griseum*	CBS 961.69	AY856884	AY916474	–	–
*N.griseum*	CBS 113542	AY916088	AY916475	–	–
*N.griseum*	UAMH 1694	AY856902	AY916473	–	–
*N.griseum*	GZCC 23-0424	PP639467	PP626611	PP596366	–
*N.griseum*	JCM 9265	AY856889	AY916476	–	–
*N.guangxiense*	GZCC 16-0042	MF467933	MF467920	MF535246	MF535276
*N.guangxiense*	MFLUCC 17-0054	MH558875	MH558750	MH550941	MH551073
*N.guangxiense*	GZCC 16-0067	MF467930	MF467917	MF535243	MF535273
*N.guangxiense*	GZCC 16-0068	MH558874	MH558749	MH550940	MH551072
*N.guangxiense*	GZCC 16-0077	MF467931	MF467918	MF535244	MF535274
*N.guangxiense*	GZCC 16-0089	MF467932	MF467919	MF535245	MF535275
*N.guangxiense*	MFLUCC 17-0050	MF467934	MF467921	MF535247	MF535277
*N.guangxiense*	MFLUCC 17-1522^T^	MF467935	MF467922	MF535248	MF535278
*N.guangxiense*	CBS 257.59	AY916087	AY916471	–	–
*N.guangxiense*	GZCC 23-0520	PP639468	PP626612	PP596367	–
*N.guineensis*	ZHKUCC 24-0113^T^	PP860102	PP860090	PP858062	PP858074
*N.guineensis*	ZHKUCC 24-0114	PP860103	PP860091	PP858063	PP858075
***N.guizhouense***	**GZCC 23-0023**	**PQ098524**	**PQ098487**	**PQ816252**	**PQ816247**
***N.guizhouense***	**GZCC 23-0078^T^**	**PQ098525**	**PQ098488**	**PQ816253**	**PQ816248**
***N.guizhouense***	**GZCC 23-0545**	**PQ098526**	**PQ098489**	**PQ816254**	**PQ816249**
*N.hainanense*	CGMCC 3.25548^T^	PP639469	PP626613	PP596368	PP596491
*N.hyalosporum*	GZCC 16-0063	MH558876	MH558751	MH550942	MH551074
*N.hyalosporum*	GZCC 16-0076^T^	MF467936	MF467923	MF535249	MF535279
*N.hyalosporum*	GZCC 22-2173	PP639470	PP626614	PP596369	PP596492
*N.hyalosporum*	GZCC 22-2162	PP639471	PP626615	PP596370	PP596493
*N.hyalosporum*	GZCC 23-0042	PP639472	–	–	–
*N.hyalosporum*	GZCC 23-0226	PP639473	PP626617	PP596371	PP596494
*N.hyalosporum*	GZCC 23-0138	PP639474	PP626618	PP596372	PP596495
*N.irregulare*	MFLUCC 17-1796^T^	MH558877	MH558752	MH550943	MH551075
*N.irregulare*	MFLUCC 17-1808	MH558878	MH558753	MH550944	MH551076
*N.jianfenglingense*	CGMCC 3.25566^T^	PP639475	PP626619	PP596373	PP596496
*N.krabiense*	MFLUCC 16-0224^T^	MH558879	MH558754	MH550945	MH551077
*N.latisporum*	CGMCC 3.25546^T^	PP639476	PP626620	PP596374	PP596497
*N.latisporum*	GZCC 22-2056	PP639477	PP626621	PP596375	–
*N.latisporum*	GZCC 22-2089	PP639478	PP626622	PP596376	–
*N.laxisporum*	GZCC 23-0224	PP639479	PP626623	–	–
*N.laxisporum*	MFLUCC 17-2027^T^	MH558880	MH558755	MH550946	MH551078
*N.morganii*	CBS 281.54	AY856876	AY916468	–	–
*N.morganii*	CBS 222.58	AY856880	AY916469	–	–
*N.ovoideum*	GZCC 16-0064^T^	MH558881	MH558756	MH550947	MH551079
*N.ovoideum*	GZCC 16-0066	MH558882	MH558757	MH550948	MH551080
*N.parvisporum*	GZCC 16-0078	MF467937	MF467924	MF535250	MF535280
*N.parvisporum*	GZCC 16-0100	MF467938	MF467925	MF535251	MF535281
*N.parvisporum*	MFLUCC 16-0218	MF467940	MF467927	MH550955	MH551087
*N.parvisporum*	MFLUCC 17-1523^T^	MF467939	MF467926	MF535252	MF535282
*N.parvisporum*	MFLUCC 17-1793	MH558884	MH558759	MH550950	MH551082
*N.parvisporum*	MFLUCC 17-1804	MH558885	MH558760	MH550951	MH551083
*N.parvisporum*	MFLUCC 17-1807	MH558886	MH558761	MH550952	MH551084
*N.parvisporum*	MFLUCC 17-1995	MH558887	MH558762	MH550953	MH551085
*N.parvisporum*	MFLUCC 17-2010	MH558888	MH558763	MH550954	MH551086
*N.parvisporum*	GZCC 22-2158	PP639480	PP626624	PP596377	PP596498
*N.parvisporum*	GZCC 23-0248	PP639481	PP626625	–	–
*N.parvisporum*	GZCC 23-0087	PP639482	PP626626	PP596378	PP596499
*N.parvisporum*	MFLUCC 17-1521	MH558883	MH558758	MH550949	MH551081
*N.suae*	CGMCC 3.23541^T^	OP184068	OP184079	OP186052	–
*N.submersum*	CBS 189.95	AY856882	AY916472	–	–
*N.submersum*	MFLUCC 17-2376^T^	MN913738	MT627738	–	–
*N.submersum*	GZCC 23-0293	PP639483	PP626627	–	–
*N.taiwanense*	BCRC-FU30841^T^	–	LC316603	–	–
*N.terrestris*	MFLUCC 23-0234^T^	PP800330	OR206384	OR206052	PP840926
*N.thailandicum*	MFLUCC 16-0221^T^	MF467941	MF467928	MF535253	MF535283
***N.wuzhishanense***	**GZCC 23-02^7^8**T	**PQ098523**	**PQ098486**	**PQ816251**	–
***N.wuzhishanense***	**GZCC 23-0279**	**PQ098527**	**PQ098490**	–	–
***N.wuzhishanense***	**GZCC 23-0326**	**PQ098522**	**PQ098485**	**PQ816250**	**PQ816246**
*N.xishuangbannaensis*	ZHKUCC 24-0119^T^	PP860104	PP860092	PP858064	PP858076
*N.xishuangbannaensis*	ZHKUCC 24-0120	PP860105	PP860093	PP858065	PP858077

Note: Newly generated sequences are in bold black. A dash (“-”) indicates unavailable data in GenBank, and “^T^” represents ex-type strains.

The maximum likelihood (ML) tree was constructed using the IQ-Tree web server (http://iqtree.cibiv.univie.ac.at/, Nguyen et al. 2015). Bayesian inference (BI) was performed following the methods described by Ma et al. (2022). The best-fit substitution model of the LSU, ITS, *tef*1-α, and *rpb*2 datasets was determined using MrModeltest v. 2.3 under the Akaike Information Criterion (AIC) (Nylander et al. 2008).

Phylogenetic trees were visualized using FigTree v. 1.4.4 and edited with Adobe Illustrator CC 2019 v. 23.1.0 (Adobe Systems, USA). Photo-plates were prepared using Adobe Photoshop CC 2019 (Adobe Systems, USA), and measurements were made using the Tarosoft (R) Image Frame Work program v. 1.3.4.

## ﻿Phylogenetic results

**Figure 1.**
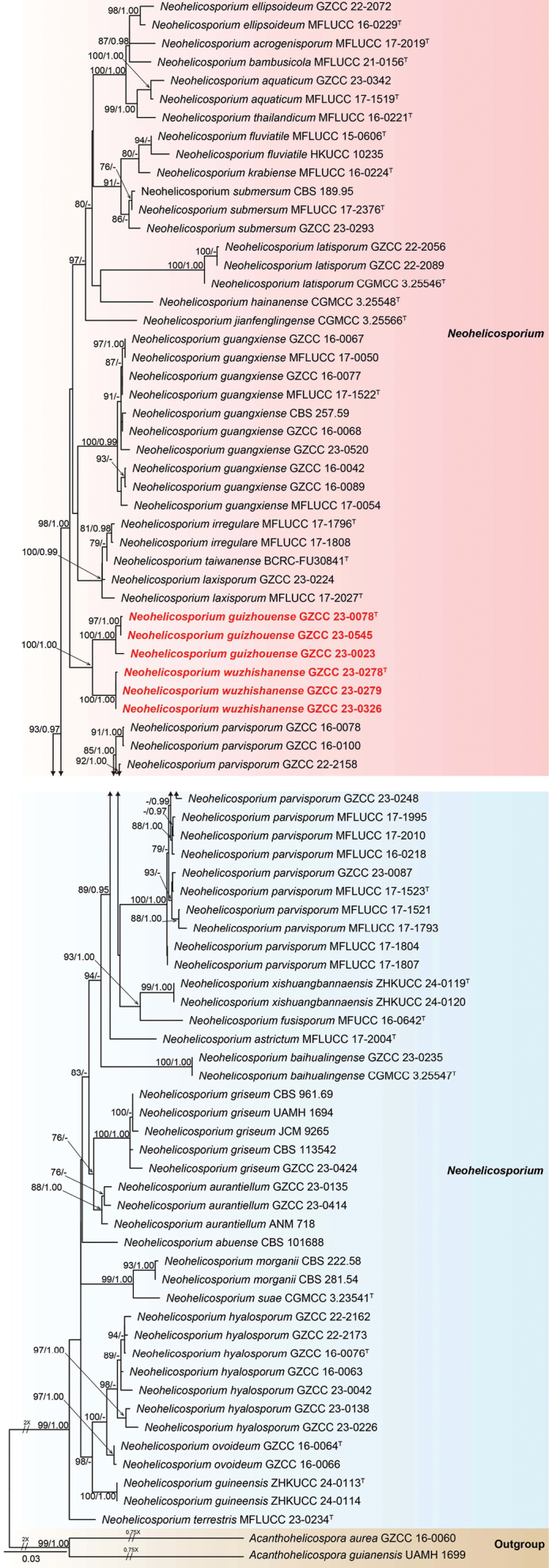
Phylogenetic tree generated from the RAxML analysis based on the combined dataset of LSU, ITS, *tef*1-α, and *rpb*2 sequences. Bootstrap support values for ML (≥ 75%) and posterior probability values (PP) (≥ 0.95) are indicated near respective nodes. *Acanthohelicosporaaurea* (GZCC 16-0060) and *A.guianensis* (UAMH 1699) were selected as outgroup taxa. Ex-type strains are indicated by “^T^”, and newly obtained isolates are in bold red font. A dash (“-”) indicates bootstrap support values below 75% for ML and PP values less than 0.95.

The phylogenetic placements of our six isolates were validated based on a multi-gene phylogenetic analysis incorporating ITS, LSU, *tef*1-α, and *rpb*2 sequence data. A total of 84 Tubeufiaceae strains, including isolates obtained in this study and two outgroups, *Acanthohelicosporaaurea* (GZCC 16-0060) and *A.guianensis* (UAMH 1699), were analyzed. The concatenated sequence matrix contains 3,386 characters (LSU: 1–842, ITS: 843–1,429, *tef*1-α: 1,430–2,341, *rpb*2: 2,342–3,386). Fig. 1 illustrates the best-scoring RAxML tree, with a final likelihood value of -15233.387. Following the guidelines of Chethana et al. (2021), a biphasic approach incorporating both morphological and phylogenetic species concepts was employed to describe the new species.

Our collections in our multi-gene phylogenetic tree (Fig. 1) represent two distinct novel *Neohelicosporium* species within Tubeufiaceae. Our isolates, GZCC 23–0078 and GZCC 23–0545, cluster together as a sister group to GZCC 23–0023, with 100% ML and 1.00 PP support. Additionally, GZCC 23–0278, GZCC 23–0279, and GZCC 23–0326 group together, forming a distinct lineage with *N.guizhouense* (GZCC 23–0023, GZCC 23–0078, and GZCC 23–0545), supported by 100% ML and 1.00 PP.

### ﻿Taxonomy

#### ﻿ Neohelicosporium guizhouense

Taxon classificationFungi

X.Y. Ma, J. Ma & Y.Z. Lu sp. nov.

BBAA5E13-348B-5187-8619-BBD3E357F368

903462

Facesoffungi Number: FoF17243

Fig. 2

##### Etymology.

The epithet “*guizhouense*” refers to Guizhou Province, where the fungus was collected.

##### Holotype.

HKAS 128908

##### Description.

***Saprobic*** on decaying wood in terrestrial habitats. **Sexual morph** Undetermined. **Asexual morph** Hyphomycetous, helicosporous. ***Colonies*** on natural substrate superficial, effuse, gregarious, white. ***Mycelium*** mostly immersed, partly superficial, composed of pale brown to brown, branched, septate, guttulate, smooth. ***Conidiophores*** 56.5–165 μm long, 4–6.5 μm wide (x̄ = 118.5 × 5.5 μm, n = 20), macronematous, mononematous, procumbent, aggregated, cylindrical, tapering towards the tip, straight or slightly flexuous, branched, septate, smooth- and thick-walled, brown at the base and hyaline towards the apex. ***Conidiogenous cells*** 9–16.5 μm long, 3.5–6 μm wide (x̄ = 12.5 × 4.5 μm, n = 30), holoblastic, mono- to poly-blastic, integrated, intercalary or terminal, hyaline, smooth-walled, cylindrical or subcylindrical, becoming truncate towards the apex after conidial secession with tiny tooth-like protrusions, mostly bearing one (rarely two) tiny conidiogenous loci. ***Conidia*** solitary, acropleurogenous, helicoid, tapering towards the ends, developing on tooth-like protrusions, 20.5–27.5 μm diam. and conidial filament 3.5–5 μm wide (x̄ = 23.5 × 4 μm, n = 30), 78–109.5 μm long (x̄ = 88 μm, n = 30), indistinctly multi-septate, slightly constricted at septa, tightly coiled up to 3 times, becoming loosely coiled or uncoiled in water, guttulate, hyaline, smooth-walled.

**Figure 2.**
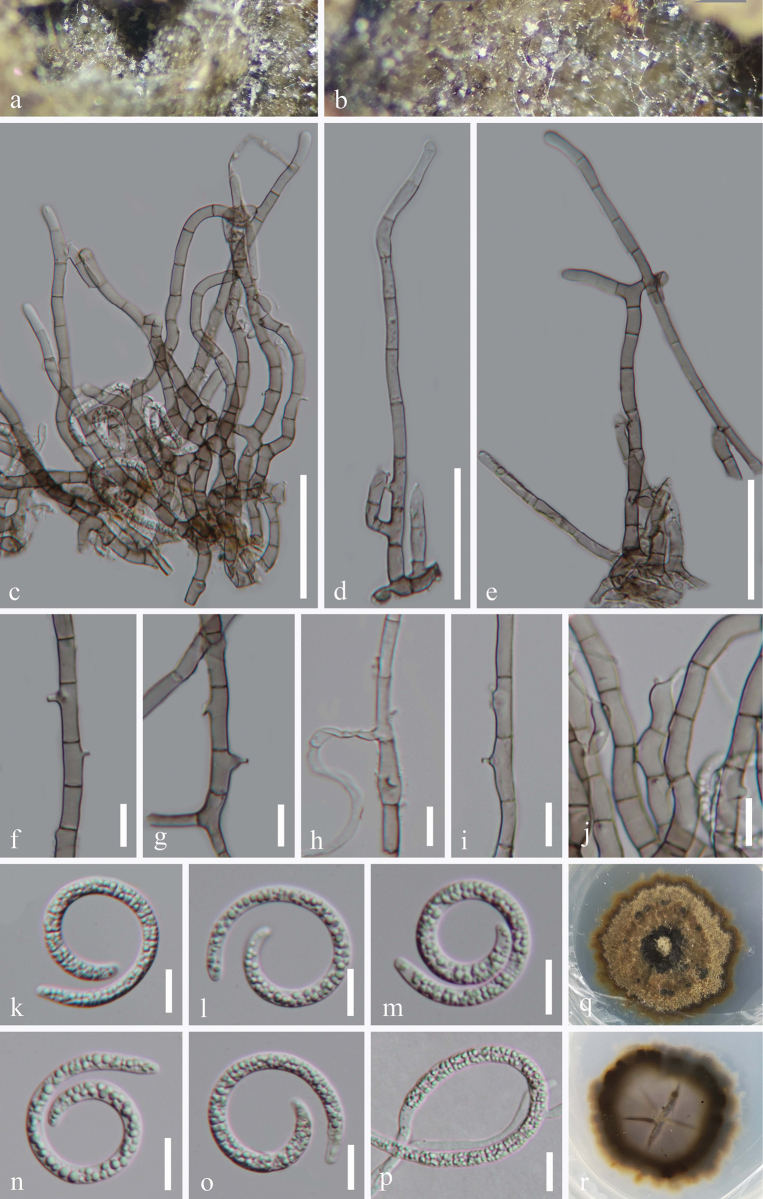
*Neohelicosporiumguizhouense* (HKAS 128908, holotype) **a, b** Colonies on the natural substrate **c–e** Conidiophores and conidiogenous cells **f–j** Conidiogenous cells **k–o** Conidia **p** Germinated conidium **q, r** Surface and reverse view of colonies on PDA after 33 days of incubation at 25 °C. Scale bars: 50 μm (**c**); 40 μm (**d, e**); 10 μm (**f–p**).

##### Culture characteristics.

Conidia germinating on PDA within 13 h and germ tubes arising from the terminal ends and the middle of the conidium. Colonies reached 26 mm diam. after 33 days of incubation at 25 °C, irregular, with flat, brown to black mycelia on the surface, in reverse pale brown to black with undulate margin.

##### Material examined.

China • Guizhou Province, Qiannan Buyi and Miao Autonomous Prefecture, Libo County, Maolan Nature Reserve, on decaying wood in a terrestrial habitat, 10 April 2022, Jian Ma, MN4.1 (HKAS 128908, holotype), ex-type living culture GZCC 23-0078; China, Guizhou Province, Sandu Shui Autonomous County, Yao Renshan National Forest Park, on decaying wood in a terrestrial habitat, 10 April 2022, Jian Ma, YS17.4 (GZAAS 23-0550, paratype), living culture GZCC 23-0545; China, Hainan Province, Ledong Li Autonomous County, Jianfengling National Forest Park, on decaying wood in a forest, 28 December 2021, Jian Ma, JB2 (HKAS 128876, paratype), living culture GZCC 23-0023.

##### Notes.

In the phylogenetic tree (Fig. 1), three strains of *Neohelicosporiumguizhouense* (GZCC 23-0078, GZCC 23-0023, and GZCC 23-0545) formed a distinct lineage and were sister to *N.wuzhishanense* (GZCC 23-0278, GZCC 23-0279, and GZCC 23-0326), supported by 100% ML and 1.00 PP. A comparison of LSU, ITS, *tef*1-α, and *rpb*2 sequences between the ex-type strain of *N.guizhouense* (GZCC 23-0078) and *N.wuzhishanense* (GZCC 23-0326) revealed 2/837 bp (0.2%, including one gap), 26/773 bp (3.4%, including one gap), 21/885 bp (2.4%, without a gap), and 35/914 bp (3.8%, including one gap) nucleotide base differences, respectively, which strongly support them as two distinct species. Morphologically, *Neohelicosporiumguizhouense* (HKAS 128908) differs from *N.wuzhishanense* (HKAS 128903) by its distinct conidiogenous cells (tooth-like vs. tooth-like and/or bladder-like), smaller conidial filaments (3.5–5 μm vs. 4.5–6 μm), and different coiled states in water (loosely coiled vs. tightly coiled). Therefore, we designate the three isolates (GZCC 23-0023, GZCC 23-0078, and GZCC 23-0545) as a new species, *Neohelicosporiumguizhouense*.

#### ﻿ Neohelicosporium wuzhishanense

Taxon classificationFungi

X.Y. Ma, J. Ma & Y.Z. Lu sp. nov.

5A9891C3-7491-537F-AA99-390117570EC5

903463

Facesoffungi Number: FoF17244

Fig. 3

##### Etymology.

The epithet “*wuzhishanense*” refers to Wuzhishan City, Hainan Province, where the fungus was collected.

##### Holotype.

HKAS 128903

##### Description.

***Saprobic*** on submerged decaying wood in freshwater habitats. **Sexual morph** Undetermined. **Asexual morph** Hyphomycetous, helicosporous. ***Colonies*** on natural substrate superficial, effuse, solitary, scattered, or gregarious, white to pale brown. ***Mycelium*** mostly superficial, partly immersed, composed of pale brown to brown, branched, septate, guttulate, smooth, with mass glistening conidia. ***Conidiophores*** 75.5–203 μm long, 5.5–6.5 μm wide (x̄ = 134.5 × 6 μm, n = 25), macronematous, mononematous, erect, solitary, cylindrical, straight or slightly flexuous, occasionally branched, septate, smooth-walled, thick-walled, wider at the base and narrower towards the apex, and brown at the base, becoming hyaline to pale brown towards the apex. ***Conidiogenous cells*** 13–29 μm long, 3.5–5 μm wide (x̄ = 19 × 4.5 μm, n = 25), holoblastic, mono- to poly-blastic, integrated, intercalary or terminal, determinate, hyaline to brown, smooth-walled, cylindrical, truncate at the apex after conidial secession, with tiny tooth-like and/or bladder-like protrusions (7–16 μm long, 3–6 μm wide (x̄ = 10.5 × 4.5 μm, n = 15)). ***Conidia*** solitary, acropleurogenous, helicoid, tapering toward the ends, developing on tooth-like or bladder-like protrusions, 20.5–28.5 μm diam. and conidial filament 4.5–6 μm wide (x̄ = 25 × 5 μm, n = 30), 92.5–138 μm long (x̄ = 118 μm, n = 30), indistinctly multi-septate, slightly constricted at septa, tightly coiled 2½–3½ times, not becoming loose in water, guttulate, hyaline, smooth-walled.

**Figure 3.**
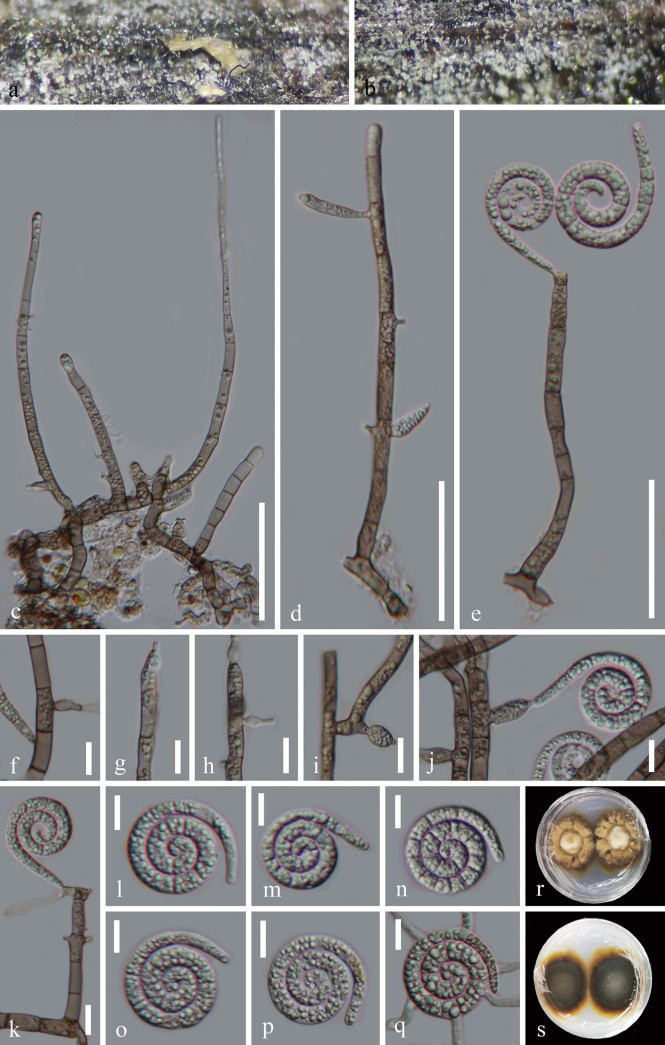
*Neohelicosporiumwuzhishanense* (HKAS 128903, holotype) **a, b** Colonies on the natural substrate **c–e, k** Conidiophores, conidiogenous cells, and attachment of conidia **f–j** Conidiogenous cells and attachment of conidia **l–p** Conidia **q** Germinated conidium **r, s** Surface and reverse view of colonies on PDA after 41 days of incubation at 25 °C. Scale bars: 50 μm (**c**); 40 μm (**d, e**); 10 μm (**f–q**).

##### Culture characteristics.

Conidia germinating on PDA within 18 h and germ tubes arising from the middle of the conidium. Colonies reached 37 mm diam. after 41 days of incubation at 25 °C, irregular, with raised, white to brown mycelia on the surface, in reverse brown to black-brown, with undulate margin.

##### Material examined.

China • Hainan Province, Wuzhishan City, Wuzhishan National Nature Reserve, on decaying wood in a freshwater stream, 28 December 2021, Jian Ma, WS68 (HKAS 128903, holotype), ex-type living culture GZCC 23-0326; • Ibid., WS19 (GZAAS 23-0282, paratype), living culture GZCC 23-0278; • Ibid., WS20 (GZAAS 23-0283), living culture GZCC 23-0279.

##### Notes.

Morphologically, *Neohelicosporiumwuzhishanense* (HKAS 128903) closely resembles *N.latisporum* (HKAS 128955) in having macronematous, mononematous, erect, cylindrical, septate conidiophores; holoblastic, monoblastic, or polyblastic, integrated, cylindrical conidiogenous cells; and solitary, acrogenous, helicoid conidia (Ma et al. 2024b). However, based on multi-gene phylogenetic analyses, *Neohelicosporiumwuzhishanense* formed a sister clade with *N.guizhouense*, which is phylogenetically distant from *N.latisporum* (Fig. 1). Morphologically, *Neohelicosporiumwuzhishanense* (HKAS 128903) differs from *N.latisporum* (HKAS 128955) by its unique conidiogenous cells (tooth-like and/or bladder-like vs. tooth-like) and longer conidia (92.5–138 μm vs. 48.5–67.5) (Ma et al. 2024b). Based on morphological characteristics and DNA sequence data, we identified GZCC 23-0278, GZCC 23-0279, and GZCC 23-0326 as a new species, *Neohelicosporiumwuzhishanense*.

## ﻿Discussion

Including the newly described species (*N.guizhouense* and *N.wuzhishanense*), the genus *Neohelicosporium* comprises a total of 35 species (Samarakoon et al. 2024; Ma et al. 2024b). Among these, 15 species have been found in terrestrial habitats, 12 species in freshwater habitats, and eight species have been reported from both freshwater and terrestrial habitats (Morgan 1892; Penzig 1897; Linder 1929; Moore 1954; Rao and Rao 1964; Matsushima 1971, 1980; Rossman 1979, 1987; Chouhan and Panwar 1980; Goos 1985, 1986, 1989; Ho et al. 2001; Tsui et al. 2001, 2006; Cai et al. 2006; Zhao et al. 2007; da Cruz et al. 2009; Promputtha and Miller 2010; Singh and Singh 2016; Jayasiri et al. 2017; Kuo and Goh 2018; Lu et al. 2018a, 2018b; Dong et al. 2020; Li et al. 2022; Tian et al. 2022; Yang et al. 2023; Zhang et al. 2023a, 2023b; Samarakoon et al. 2024; Ma et al. 2023, 2024b). These species occur as saprobes on bamboo, *Elaeisguineensis*, Malvaceae sp., *Musa* sp., and decaying wood of unknown origin (Jayasiri et al. 2017; Lu et al. 2018a, b; Tian et al. 2022; Ma et al. 2024a; Samarakoon et al. 2024; Xiong et al. 2024).

Significant intra-species morphological variations have been observed within *Neohelicosporium*. For example, two collections (HKAS 128940 from submerged decaying wood in China and MFLU 17-1734 from submerged decaying wood in Thailand) represent the same species, *Neohelicosporiumsubmersum* (Dong et al. 2020; Ma et al. 2024b). However, HKAS 128940 exhibits shorter conidiophores (38–77 µm) compared to MFLU 17-1734 (50–260 µm). Furthermore, the conidiogenous cells in HKAS 128940 are sympodial, a feature not observed in MFLU 17-1734 (Dong et al. 2020; Ma et al. 2024b). Similarly, *Neohelicosporiumlaxisporum* (MFLU 17-1107 and GZAAS 23-0228), collected from terrestrial habitats in China and Thailand, display notable morphological differences. GZAAS 23-0228 has wider conidiophores (4.5–8 μm vs. 3.5–5 μm) and wider conidiogenous cells (4.5–6 μm vs. 3–4 μm) than MFLU 17-1107 (Lu et al. 2018b; Ma et al. 2024b). Additionally, the conidiogenous cells of MFLU 17-1107 consist of tiny tooth-like protrusions, whereas GZAAS 23-0228 exhibits tiny tooth-like protrusions as well as bladder-like protrusions (Lu et al. 2018b; Ma et al. 2024b). These morphological variations are likely due to geographical differences.

The conidiophores and conidia of some *Neohelicosporium* species closely resemble those of *Parahelicomyces* and *Tubeufia* (Lu et al. 2018b; Li et al. 2022; Ma et al. 2024b). *Neohelicosporiumlatisporum* (HKAS 128955) resembles *Parahelicomycesparvisporus* (HKAS 128868) in having erect, solitary conidiophores and wider, tightly coiled, brown conidia (Jayasiri et al. 2017; Kuo and Goh 2018; Lu et al. 2018a, 2018b; Dong et al. 2020; Li et al. 2022; Tian et al. 2022; Yang et al. 2023; Samarakoon et al. 2024; Ma et al. 2023, 2024b). In addition, five *Neohelicosporium* species, namely *N.ellipsoideum* (GZAAS 22-2072), *N.hainanense* (HKAS 128921), *N.jianfenglingense* (HKAS 128914), *N.suae* (HKAS 124610), and *N.submersum* (HKAS 128940), morphologically resemble *Tubeufia* in having short, simple, erect, irregularly cylindrical conidiophores (Lu et al. 2017, 2018a, 2018b; Li et al. 2022; Ma et al. 2023, 2024b). These morphological similarities highlight the challenges of distinguishing genera based solely on morphology, emphasizing the importance of molecular and phylogenetic analyses for accurate identification and delineation.

## Supplementary Material

XML Treatment for Neohelicosporium guizhouense

XML Treatment for Neohelicosporium wuzhishanense

## ﻿Additional information

### Author ORCIDs

Yong-Zhong Lu https://orcid.org/0000-0002-1033-5782

Jian Ma https://orcid.org/0009-0008-1291-640X

### Data availability

All of the data that support the findings of this study are available in the main text.
